# Torsades de pointes during laparoscopic adrenalectomy of a pheochromocytoma: a case report

**DOI:** 10.1186/1752-1947-5-368

**Published:** 2011-08-12

**Authors:** Kinge van der Heide, Ann de Haes, Götz JK Wietasch, Ans CP Wiesfeld, Herman GD Hendriks

**Affiliations:** 1 Department of Emergency Medicine, University Medical Centre Groningen, PO Box 30.001, 9700 RB Groningen, The Netherlands; 2 Department of Anaesthesiology, Rijnstate Ziekenhuis, PO Box 9555, 6800 TA Arnhem, The Netherlands; 3 Department of Anaesthesiology, University Medical Centre Groningen, PO Box 30.001, 9700 RB Groningen, The Netherlands; 4 Department of Cardiology, University Medical Centre Groningen, PO Box 30.001, 9700 RB Groningen, The Netherlands

## Abstract

**Introduction:**

Torsades de pointes is a rare but potentially lethal arrhythmia. The amount of literature available on Torsades de pointes occurring in patients with pheochromocytoma is limited, and we found no literature describing this dysrhythmia in a patient with pheochromocytoma under anesthesia.

**Case presentation:**

We describe the case of a 42-year-old Caucasian woman without QT prolongation preoperatively with recurrent Torsades de pointes during laparoscopic removal of a pheochromocytoma. Torsades de pointes mainly occurs in the setting of a prolonged QT interval. This patient neither had a prolonged QT preoperatively nor was her family history suspect for a congenital long QT syndrome. Most likely, our patient had an acquired long QT syndrome, elicited by the combination of flecainide, hypomagnesemia and adrenergic stimulation during manipulation of the tumor.

**Conclusion:**

We show that in the case of a surgical pheochromocytoma removal, perioperative conditions can elicit an acquired or previously unknown congenital long QT syndrome. Therefore, preoperative α- and β-blockade is advised, QT-prolonging drugs should be avoided and potassium and magnesium plasma levels should be kept at normal to high levels.

## Introduction

Pheochromocytomas are catecholamine-producing neuroendocrine tumors arising from the chromaffin cells of the adrenal medulla or extraadrenal paraganglia. A pheochromocytoma is a potential life-threatening disease with a high risk of cardiovascular complications such as myocardial infarction, arrhythmias, catecholamine-induced cardiomyopathy, stroke and pulmonary edema. It is a rare neoplasm, occurring in one to two per 1000 patients with hypertension. The relatively high prevalence of pheochromocytoma in autopsy studies (0.05%) indicates that the diagnosis is often missed [1]; the overall incidence is estimated to be 1.6 to 8 cases per million inhabitants per year [1].

Traditionally, adrenalectomy for pheochromocytoma has been performed by open lateral retroperitoneal surgery [2]. Nowadays, laparoscopic removal of intraadrenal and extraadrenal pheochromocytomas is the preferred surgical treatment because it reduces postoperative morbidity, hospital stay and costs compared with conventional laparotomy [1]. Induction of general anesthesia and surgical tumor manipulation are the most well-known stimuli to evoke an acute catecholaminergic crisis. About 25% to 50% of hospital deaths of patients with pheochromocytoma occur during surgery [3]. This report describes torsades de pointes (TdP) in a patient during laparoscopic removal of a pheochromocytoma as a rare perioperative complication.

## Case presentation

A 42-year-old Caucasian woman was referred to our university hospital because of a pheochromocytoma of the left adrenal gland. For one year, she had experienced episodic headaches, palpitations, sweating, chest discomfort, orthostatic hypotension and fatigue. A computed tomography scan showed a large adrenal mass, and urine and blood tests confirmed the diagnosis of a catecholamine-producing mass (mainly epinephrine and norepinephrine in a lesser degree). For symptomatic treatment, combined α- and β-blockade was started with doxazosin (8 mg once daily per os [PO]) and propranolol (20 mg thrice daily PO). Because electrocardiography showed atrial fibrillation, the patient was also treated with flecainide (100 mg once daily PO) and acenocoumarol (PO; INR conducted, 2.5-3.5).

**Figure 1:**
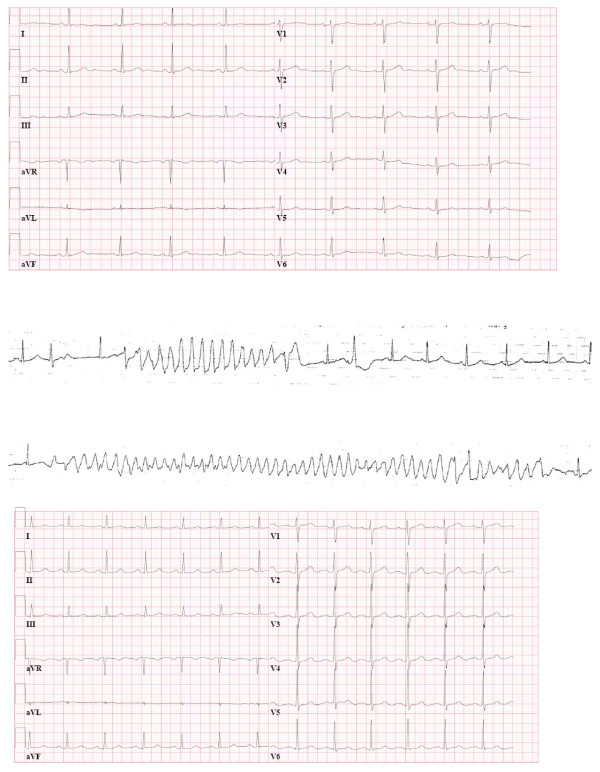
**Perioperative electrocardiographic (ECG) recordings**. **a) **Preoperative ECG. Sinus rhythm, 58 beats/min. Without QT prolongation (QTc, 440 msec). **b) **Two recordings of torsades de pointes episodes. Note the short-long-short phenomenon preceding the arrhythmia in the upper recording. **c) **Postoperative ECG. Sinus rhythm, 77 beats/min. With prolonged QT interval (QTc, 505 msec), best seen in aVF. In leads V1-V4, there are possible U waves merging into the P wave.

Preoperative examination revealed a blood pressure of 130/75 mm Hg in the supine position (120/85 mm Hg standing upright) under combined α- and β-blockade. At admission, she had a sinus bradycardia of 58 beats/min with a normal QRS width (110 msec) without prolonged corrected QT interval (QTinterval corrected for heart rate (QTc), 435 msec) (Figure 1a).

Blood count and serum electrolytes were within normal ranges, but her serum magnesium level was not determined.

Anesthesia was performed with propofol (2.5 mg/kg intravenously [IV]), sufentanil (0.4 μg/kg IV) and rocuronium (0.6 mg/kg IV). Isoflurane (1.1%-1.3%) was used for maintenance along with increments of sufentanil and rocuronium when appropriate. A central venous line (16-Fr, double-lumen, right internal jugular vein), an arterial line (20 gauge; left radial artery) and two peripheral IV lines (14 and 20 gauge) were inserted. To perform a left laparoscopic adrenalectomy, the patient was placed in a right lateral position. The patient's blood pressure and heart rate remained stable during induction and positioning.

Immediately after surgical manipulation of the tumor, the patient's blood pressure increased from 155/95 to 200/105 mm Hg. To control hypertension, nitroprusside (25 μg/kg/hr IV) was started, and an extra propofol bolus (100 mg IV) was administered. Despite these measures, the second surgical manipulation of the tumor resulted in a blood pressure of 245/110 mm Hg immediately followed TdP (Figure 1b). On request of the anesthetist, the surgeons stopped manipulating the tumor, resulting in a spontaneous return toward sinus rhythm within a few seconds and a gradually normalizing of the blood pressure. However, each time the surgeons tried to ligate the tumors venous return, the blood pressure elevated. Nitroprusside was increased to 130 μg/kg/hr, and two esmolol boluses of 20 mg each were administered. Also, anesthesia was deepened with propofol (100 mg thrice) and two sufentanil boluses of 20 μg and 10 μg. Despite these interventions, the patient's blood pressure did not decrease below diastolic blood pressure of 105 mm Hg. At each blood pressure peak (maximum, 254/112 mm Hg), the patient showed TdP. In total, she experienced this arrhythmia four times, all returning to sinus rhythm.

The total period of arrhythmias lasted 15 minutes and ended abruptly when the venous return of the tumor was ligated. Subsequently, the tumor was successfully removed. Serum electrolytes were tested directly after removal of the tumor and revealed slight hypomagnesaemia (0.54 mmol/L), normokalemia and normal sodium. Pupillary reflexes were found normal during the whole procedure. To prevent hypotension after tumor ligation, the patient was administered IV norepinephrine (10 mg/50 mL), which could be stopped at the end of the operation. The postoperative electrocardiogram (ECG) showed sinus rhythm with 77 beats/min, but in contrast to the preoperative ECG, now with prolonged QT interval (QTc 505 msec) and U waves (Figure 1c). Her postoperative stay was uneventful. Pathologic examination confirmed the diagnosis of a pheochromocytoma.

## Discussion

We describe the case of a patient without QT prolongation preoperatively with recurrent TdP during laparoscopic removal of a pheochromocytoma. TdP is a form of polymorphic ventricular tachycardia, predominantly occurring in the setting of a prolonged QT interval, T-wave abnormality or increased U-wave amplitude [4]. It occurs frequently in the presence of severe bradycardia and often precedes ventricular fibrillation. Electrocardiographically, TdP is a pattern of continuously changing morphology of the QRS complexes twisting around an imaginary baseline. In most cases, including in our patient, TdP is preceded by a characteristic sequence of a long RR interval followed by a short extrasystolic interval with premature depolarization interrupting the preceding repolarization, called the short-long-short phenomenon (Figure 1b).

The QT interval represents the depolarization and repolarization of the ventricles [5-8]. Prolongation of the QT interval is caused by an increase in action potential duration of ventricular myocytes [6-8]. The ventricular myocardium is predominantly composed of three cell types that are histologically alike yet vary electrophysiologically and pharmacologically. These three cell types may respond differently to drug- or disease-mediated action potential prolongation and hence differences in repolarization ("inhomogeneous prolongation of repolarization"). Transmural dispersion of repolarization may occur [7], which may be considered the electrophysiologic cause of TdP. Transmural dispersion of repolarization may be increased by an adrenergic agent such as isoproterenol [6-8].

In contrast to the patient's normal preoperative QT interval, the postoperative QT interval was prolonged. A QT interval can be prolonged congenitally or acquired. Congenital long QT syndrome (LQTS) is subdivided into 10 genotypes. In LQT1 and LQT2, cardiac events may be precipitated by physical or emotional stress. These patients are treated with antiadrenergic therapy, such as β-blockers. Identification of the congenital LQTS genes uncovered groups of patients with normal resting ECGs. It is thought that these patients have an incomplete penetrance and that they are mutation carriers or are carrying polymorphic congenital LQTS disease genes. They are at risk of developing TdP when exposed to certain drugs [5]. Nothing, however, is known about adrenergic stimulation in this group of patients. An "epinephrine stress test" is sometimes performed to unmask this group of patients with a supposed congenital LQTS and a normal QT interval. In this provocative test, epinephrine is administered IV by bolus infusion (Shimizu protocol) or by incremental escalating infusion (Mayo protocol) while ECG changes are measured. A paradoxical response characterized by QT lengthening (rather than expected shortening) is seen more frequent in patients with LQT1 [9].

Our patient neither had a prolonged QT preoperatively, nor was her family history suspect for a congenital LQTS. Moreover, in a follow-up study, her exercise test and echocardiography results were also normal, and a 24-hour ambulatory monitoring only showed a short episode of a spontaneous supraventricular tachycardia, probably originated by atrial tachycardia. Possibly, she had a LQTS1 during surgery originated by excess of adrenergic stimulation. We did not perform genetic testing afterward to confirm this because noninvasive cardiac evaluation results were normal, and her family history results were negative. Most likely, our patient experienced an acquired LQTS. An acquired LQTS is predominantly elicited by drugs prolonging the ventricular action potential or by an electrolyte imbalance [10]. In the present case, the combination of flecainide [10], hypomagnesaemia and adrenergic stimulation may have elicited increased transmural dispersion of repolarization, resulting in TdP [6-8]. The increased adrenergic stimulation during manipulation of the tumor then likely resulted in premature ventricular beats either by abnormal automaticity or by early afterdepolarizations inducing a pause (long RR interval) and increased transmural dispersion of depolarization resulting in TdP. This is, however, easily said in a retrospective point of view. The choice of using flecainide was clear because there was atrial fibrillation, and the plasma magnesium level is not a standard preoperative measurement.

Anesthetic guidelines on managing patients with pheochromocytoma stress the importance of preoperative treatment with α-blocking and, if necessary, with β-blocking agents. Often, phenoxybenzamine or doxazosin is advised [2,3,11]. Preoperative hemodynamic changes are preferably controlled with phentolamine, nitroprusside and short-acting β-blockers such as esmolol [2,3,11]. Because no prospective, controlled, randomized trials have been performed on almost any aspect of the diagnosis or treatment of pheochromocytoma, guidelines are based on expert opinions and case reports. TdP occurring in patients with pheochromocytoma is not expected, and specific combinations of drugs to minimize the risk of TdP are unknown. We advise, understandably, that sufficient α- and β-blockade is mandatory, but QT-prolonging drugs should also be avoided [10]. Moreover, potassium and magnesium plasma levels should be kept at normal to high levels.

## Conclusion

A laparoscopic adrenalectomy of a pheochromocytoma in a patient without preoperative QT interval prolongation may result in TdP, most likely elicited by an excess of adrenergic stimulation. Thorough preoperative α- and β-blockade is advised, QT-prolonging drugs should be avoided and potassium and magnesium plasma levels should be kept at normal to high levels.

## Consent

Written informed consent was obtained from the patient for publication of this case report and accompanying images. A copy of the written consent is available for review by the Editor-in-Chief of this journal.

## Competing interests

The authors declare that they have no competing interests.

## Authors' contributions

KvdH, AdH and HGDH were present during the event described in this case report. KvdH and AdH collected the literature and the patient data. KvdH was the major contributor in writing the manuscript. Reviewing the manuscript was mostly done by KvdH, JKGW and HGDH. ACPW was asked for her expertise on torsades de pointes and also contributed to reviewing the manuscript. All authors read and approved the final manuscript.
